# The Impact of Catch-Neuter-Vaccinate-Return (CNVR) on Greater Bangkok Residents’ Attitudes and Behaviours Towards Free-Roaming Dogs

**DOI:** 10.3390/ani15091274

**Published:** 2025-04-30

**Authors:** Elly Hiby, Tuntikorn Rungpatana, Alicja Izydorczyk, Valerie Benka, Craig Rooney

**Affiliations:** 1 International Companion Animal Management (ICAM) Coalition, Cambridge CB23 7EJ, UK; 2 Soi Dog Foundation, Phuket 83110, Thailand; tuntikorn@soidog.org (T.R.); ala@soidog.org (A.I.); 3 Independent Researcher, Ann Arbor, MI 48103, USA; vbenka@gmail.com; 4 Dogs Trust, London EC1V 7RQ, UK; craig.rooney@dogstrust.org.uk

**Keywords:** dog, free-roaming dog, stray dog, catch, neuter, vaccinate, return, CNVR, animal birth control, sterilisation, rabies, dog welfare

## Abstract

Between 2016 and 2023, over 400,000 dogs in Greater Bangkok, Thailand, were sterilised and vaccinated against rabies through a Catch, Neuter, Vaccinate, Return (CNVR) programme. CNVR is practiced in many countries to humanely and effectively manage free-roaming dog populations, and to mitigate canine and human rabies risks. Residents were surveyed about their experiences, attitudes, and behaviours towards free-roaming dogs. Responses were evaluated in relation to respondents’ dog ownership status and the CNVR intensity within their local administrative area (0, 1, 2, or 3 CNVR rounds). The results show an overall positive effect of CNVR. A dose-dependent CNVR effect was observed with residents’ perceptions of declines in free-roaming dog populations and puppies born in their community, as well as in being “troubled” by free-roaming dogs now versus before the intervention. Residents living in areas with the highest levels of CNVR also had lower odds of reporting a dog bite. Some measures, including other current attitudes and behaviours towards free-roaming dogs, did not exhibit this dose-dependent effect. Dog owners showed higher levels of tolerance and care for street dogs than non-owners. The results suggest that owned dogs contributed substantially to the roaming dog population and must be considered in management efforts. CNVR appears to have delivered meaningful benefits to Greater Bangkok residents, and the majority reported sterilisation as their preferred management approach.

## Simple Summary

Catch, Neuter, Vaccinate, Return (CNVR) is practiced in many countries to manage populations of free-roaming dogs. Greater Bangkok, Thailand, is one area in which CNVR has been used to manage roaming dog numbers, improve welfare, and mitigate the risk of rabies through the sterilisation and vaccination of more than 400,000 dogs between 2016 and 2023. A survey of over 3200 Greater Bangkok residents explored their experiences, perceptions, and behaviours towards free-roaming and owned dogs. Survey results reveal multiple ways in which the number of CNVR rounds performed within a resident’s local neighbourhood predicted positive experiences, attitudes, and behaviours toward free-roaming dogs. Findings show that residents themselves report both fewer free-roaming dogs and decreased nuisance behaviours in areas with CNVR, demonstrating that sustained CNVR programmes produce meaningful benefits to citizens. Sterilisation was also selected as the preferred management approach by the majority of respondents. These are compelling findings for policymakers considering investments in humane dog population management to improve resident and community health and well-being in areas with populations of free-roaming dogs.

## 1. Introduction

Estimates of the global dog population range from 700 million [1] to 1 billion [2]. Some of these dogs are closely supervised owned dogs who are confined to a home. Many more are owned dogs that roam freely outside the household, community dogs that have no single reference household but are offered care by several people, or unowned dogs [3]. These last three subpopulations of dogs comprise the free-roaming dog (FRD) population. The proportion of each subpopulation varies significantly by community, dependent on dog ownership practices and the availability of resources in public spaces, including the purposeful feeding of FRDs and public tolerance of their presence.

The health and welfare of FRDs can be poor [4,5,6,7]. These dogs can also have a detrimental impact on communities, including exhibiting nuisance behaviour [8,9], contributing to and being victims of vehicle collisions [8,10], endangering wildlife and livestock [6,9,11,12], causing hygiene problems with urine and faeces [9,10], and posing public health risks, including biting people and transmitting zoonotic diseases [4,6,8,13]. Rabies is of high concern due to its almost 100% fatality rate (an estimated 59,000 people die from rabies annually [14]) and exceedingly high economic costs (USD 8.6 billion) associated largely with death and post-exposure prophylaxis [14].

Due to these factors, FRDs are an ongoing focus of research, interventions, and population and rabies control efforts [9,10,15,16,17,18,19,20]. Mass annual vaccination of FRDs is the core recommended approach for preventing rabies risk to people by controlling and eliminating the canine rabies virus from the dog population [21,22,23,24]. Population control methods that have been practiced include culling (killing) dogs, often through methods that are inhumane for dogs and dangerous to other species; containing dogs in a shelter, leading to adoption, euthanasia, or long-term confinement, often in overcrowded facilities; and fertility control, typically through surgical sterilisation combined with rabies vaccination [6,13,25]. This last method, when applied to FRDs, is called Catch, Neuter, Vaccinate, Return (CNVR) or Animal Birth Control (ABC). This is a non-lethal population management and rabies prevention approach specifically for community and unowned dogs [6,11,20,26]. It entails surgically sterilising dogs, vaccinating them, and returning them to where they were picked up [20].

Evaluations of FRD population management interventions have considered multiple outcomes: improving dog welfare, improving public perception of and care provided to dogs, reducing dog population density and stabilising population turnover, improving animal shelter or rehoming centre performance, reducing risks to public health, and reducing negative impacts of dogs on wildlife and livestock [6,11]. Socioeconomic impact is a nascent area of inquiry [27].

Due to their large numbers of FRDs and high rabies burden, South and Southeast Asian countries have been a focus of efforts to manage dog populations and canine-mediated rabies. Many programmes have utilised a CNVR model [20,26,28]. Soi Dog Foundation, a Thai animal welfare organisation, has led CNVR efforts in several provinces in Thailand [29]. Between 2016 and mid-2023, and in partnership with the UK-based animal welfare organisation Dogs Trust, Soi Dog Foundation worked with local authorities to deliver CNVR services to over 400,000 dogs in Bangkok and five adjacent provinces. These six provinces together comprise Greater Bangkok.

The CNVR services in Greater Bangkok have targeted free-roaming unowned dogs and community dogs but have also welcomed owned dogs into the intervention whose owners consent to surgery [20]. CNVR has been delivered using a strategic high-intensity rotational approach [20,26,30]. This entails focusing on each district (or subdistrict, when the district covers a large area) to sterilise and vaccinate at least 80% of its FRDs before moving to the neighbouring district. Once all districts have been reached, the team returns to the first district to conduct the next “round” of CNVR, which targets dogs missed during the prior round plus dogs new to the area [20]. As of early 2025, CNVR efforts in Greater Bangkok are ongoing, with over 500,000 dogs treated [29].

The impact of CNVR in Greater Bangkok has been evaluated using multiple methods. These have included observational surveys conducted annually to estimate the changes in free-roaming dog populations; a comparison of those changes with sterilisation rates; assessment of laboratory-confirmed dog rabies cases in Greater Bangkok; and an online attitude survey conducted in 2020 to measure the public perception of FRDs. By these measures, FRD density was reduced by 25% on average over the first five years, with a dose-dependent effect of CNVR. More rounds of CNVR led to greater localised declines in FRD density, reduced dog rabies cases, and improved dog–human relationships [20]. The most recent unpublished analysis of street survey data indicates that the reduction in FRD density has increased to 60% over eight years of performing CNVR.

A Knowledge, Attitudes, and Practices (KAP) survey, conducted in July and August of 2023, is part of this comprehensive assessment of CNVR impact. It was designed to explore public attitudes and reported experiences with, and behaviours towards, FRDs. It also aimed to explore the owned dog population, including estimating the number of owned dogs in Greater Bangkok and the care provided to them.

The KAP survey complements earlier assessments and helps to fill important knowledge gaps about CNVR’s impact in Greater Bangkok. It focuses on human care of and attitudes towards both owned dogs and street dogs, which is particularly relevant given the high numbers of owned dogs who roam freely in Thailand and other countries with large numbers of FRDs [4,15,17,20,31]. It also considers various outcomes, including roaming dog numbers, care of street dogs, care of owned dogs, and dog bite incidents, in relation to the number of CNVR rounds conducted in a particular administrative area. The findings can inform the adaptation of dog population management initiatives and contribute to a more comprehensive evaluation of CNVR’s impact.

This paper reports KAP survey findings on residents’ attitudes and behaviours towards both owned dogs and FRDs in relation to the seven-year CNVR effort (including varied numbers of CNVR rounds conducted in different areas), and in relation to dog ownership status. The survey investigated the impacts of CNVR and dog ownership on people and how they interact with, tolerate, and care for dogs they own and dogs that roam in their community. A companion paper [32] focuses on Greater Bangkok residents’ attitudes and practices related specifically to owned dogs.

## 2. Materials and Methods

### 2.1. Study Area

Greater Bangkok, also known as the Bangkok Metropolitan Region, covers nearly 8000 km^2^ and includes Bangkok plus five adjacent provinces: Nakhon Pathom, Pathum Thani, Nonthaburi, Samut Prakan, and Samut Sakhon [33]. There are 50 “khets” (districts) in Bangkok, and 309 subdistricts in the other five provinces, totalling 359 similarly sized administrative areas. At the time of this study, the six provinces were home to 11 million people, with provincial populations ranging from approximately 589,000 (Samut Sakhon) to 5.5 million people (Bangkok) [34]. Bangkok has both the largest human population and highest population density, although multiple provinces are rapidly becoming more urbanised [33].

The intervention began in Bangkok and expanded out to the other provinces. Clinics experienced variation in the number and accessibility of dogs, which influenced the speed at which rounds were completed in each area. As a result, different areas of Greater Bangkok experienced different numbers of rounds, and timing patterns, of CNVR.

### 2.2. Survey Design

The survey consisted of 42 questions, 18 of which focused on FRDs (i.e., dogs not belonging to the respondent, also called “street dogs”) and dog bites. These questions on FRDs were designed to explore the changes in FRDs likely to be important to the citizens in Bangkok, and those that could feasibly have been achieved through the CNVR intervention. Street surveys conducted as part of the intervention indicated a decline in FRD density, with greater declines in those areas that had received the most CNVR attention [20]. The remaining questions asked about dogs in the household and respondent demographics [32].

Most questions were multiple-choice, sometimes with an option to provide further details following a response of “other”. For each interview, the interviewer also recorded the GPS location (latitude, longitude, altitude, and accuracy) and selected the khet or subdistrict name from a list of 100 options. Interviewers did not proactively state their Soi Dog Foundation affiliation but answered truthfully if asked. They then noted this disclosure.

The 18 questions asked respondents the following: Do they know of any puppies born in households on their soi (Thai for “street”) in the past year and, if so, what happened to those puppies? Have they been annoyed or troubled by FRDs in the past month and, if so, what was the cause? Has the household experienced any dog bites in the last year and, if so, who was bitten and by what dog? Have they ever visited a dog shelter in Thailand? What do they feel should be done about FRDs? How do they feel about the number of dogs roaming on their soi? Do they offer regular care to “street dogs” and, if so, what kind of care? Respondents were also asked if they think the number of dogs roaming on their soi has changed over two time periods: in the past year and since 2014. These two periods were used because the CNVR effort in Greater Bangkok had been active for seven years at the time of the survey, so changes over the prior year would not capture the intervention’s earlier impacts. Respondents were asked to share their perceptions of change over nine years by “landmarking” with the memorable event in 2014 when Prayut Chan-o-cha became the prime minister of Thailand. This was chosen because it is an occasion that would resonate with most, if not all, residents of Thailand and occurred shortly before this intensive CNVR effort in Bangkok began in mid-2016. The complete questionnaire is available in Supplementary File S1.

### 2.3. Informed Consent and Confidentiality

Before beginning the survey, interviewers introduced themselves by their first names. They explained that the purpose of the interview was to better understand dogs in the respondent’s community, that there were no essential questions and respondents could skip any question, and that there was neither a cost to nor compensation for participating. Interviewers also explained that the data would be shared within organisations that can provide services for dogs and with consultants to analyse the data.

Respondents were required to be at least 18 years of age to take part in the survey independently; if under 18, they could participate if supervised by an adult from within their household. Due to the scope of the questions, respondents were also required to have lived in the household for at least one year.

The survey was completed without asking respondents to identify themselves. At the end of the survey, the respondent was asked if they wanted to share their name and contact information for follow-up or remain anonymous.

### 2.4. Data Collection

The survey was conducted in Thai using face-to-face interviews between 21 July and 27 August 2023. Twenty interviewers from Soi Dog Foundation worked in pairs, entering the responses given during the interview into an app on their smartphone [35].

Respondents were selected using stratified random sampling. Administrative areas were categorised according to the number of CNVR rounds that had taken place (0, 1, 2, or 3). Twenty-five random areas were selected from each of these categories, which created a sample of 100 administrative areas for the survey. Within each sample area, 16 random GPS points were created from areas identified to be residential using High-Resolution Population Density Maps and Demographic Estimates [36]. This showed human population density as “raster cubes”, in which a density greater than one was assumed to be a residential building. A 30 m buffer was added to each cube. Sixteen random points were created along the roads that fell within these buffered populated raster cubes. A Google map link was created for each random point. The two households closest to each of these points were selected for interviews. If no one was home or the resident refused to take part, the next closest household was selected until two surveys were completed for the point. This returned a sample of approximately 800 household respondents per category of CNVR rounds. This sample size was calculated based on the assumption that 20% of households would own an average of 1.5 dogs, resulting in 240 owned dogs from which the target sterilisation and vaccination coverage of 80% could be measured with 5% error and 95% confidence. This was a core metric of interest reported in the companion paper [32], which focuses on Greater Bangkok residents’ attitudes and practices related specifically to owned dogs.

### 2.5. Data Analysis

Data were analysed using binomial Generalised Linear Mixed Models (GLMMs). This enabled testing for the “fixed” effect of the number of CNVR rounds on the binomial outcomes (e.g., reporting being happy or accepting of the number of FRDs versus choosing one of the neutral or negative response options). The khet or subdistrict was included as a “random” effect to address the possibility that respondents living in the same khet/subdistrict may share similar characteristics related to their location, and it improved the power of the analysis.

The use of GLMMs means that each number of rounds of CNVR (1, 2, or 3) was compared with the control group (0 CNVR rounds). The number of rounds of CNVR could instead have been used as a numeric predictor to test the impact of increasing rounds on the outcome variable. However, this would require the assumption that each round of CNVR had an equal impact on the outcome variable, which was unlikely.

The data were uploaded to KoboToolbox during the collection and then downloaded to Excel and analysed using R version 4.3.2 via RStudio [37,38].

### 2.6. Ethical Approval

All procedures were in accordance with the 1964 Helsinki Declaration and its later amendments or comparable ethical standards. In addition, all methods were approved by the Dogs Trust Ethical Review Board of Dogs Trust UK (reference number ERB037 on 21 October 2020).

## 3. Results

### 3.1. Respondents

A total of 3437 households were approached, and someone was home in 3304. Ninety-nine households immediately declined to take part in the survey and two declined after hearing the consent statement, which generated a 93% response rate. Among the 3203 respondents, 303 (9.5%) asked for the interviewer’s affiliation and were provided with this information.

Respondents were evenly divided between female (50.1%) and male (49.8%). Their average age was 46 years (range 13–85). Sixteen respondents (0.5%) were under 18 years old and had adult supervision during the interview.

The sampling frame ensured that the number of respondents was approximately equal between the four categories of CNVR rounds. Achieving this balance required oversampling in some provinces (Table 1), particularly Pathum Thani, to reach sufficient respondents in khets/subdistricts with no CNVR (CNVR rounds = 0).

### 3.2. Changes in Street Dog Populations and Resident Attitudes over Time

When asked about their perceived changes in street dogs over the past year, 57% of the 3038 respondents that had lived in the area for at least one year (1733 people) felt that the number of FRDs on their soi had stayed the same. Approximately one-third (34%, 1026 people) felt the number had decreased, and 9% (279 people) reported that it had increased.

**Figure 1:**
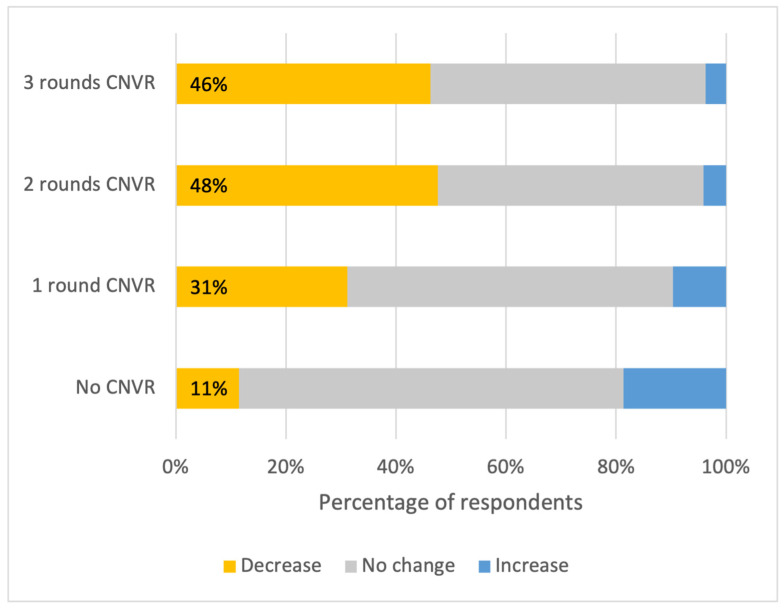
Percentage of respondents that reported a decrease, no change, or an increase in the number of FRDs on their soi over the previous year, split by the number of rounds of CNVR in their khet/subdistrict.

Respondents living in khets/subdistricts that had experienced CNVR had much higher odds of reporting a decrease in the number of street dogs on their soi in the previous year compared with respondents living in control areas (Table 2). Perceptions of population changes differed according to the numbers of CNVR rounds. Among respondents who lived in khets/subdistricts that had experienced two or three rounds of CNVR, nearly half of respondents reported that the number of street dogs had decreased. This was much greater than for areas with one CNVR round (31% perceived a decrease) and control areas (11% perceived a decrease). Control areas and those with one round of CNVR similarly had larger proportions of residents who reported an increase in dogs over the past year (Figure 1).

When asked how the number of dogs on their soi had changed over the past nine years, 35% of 3072 respondents (1083 people) agreed with the statement that there “used to be lots more dogs on my soi”. Approximately one-quarter of respondents (26%, 809 people) felt that dog numbers had remained stable over this 9-year period, and 9% (262 people) felt that dog numbers had increased in this timeframe. The remaining 30% (918) did not know or had moved to the area recently and could not assess the change.

**Figure 2:**
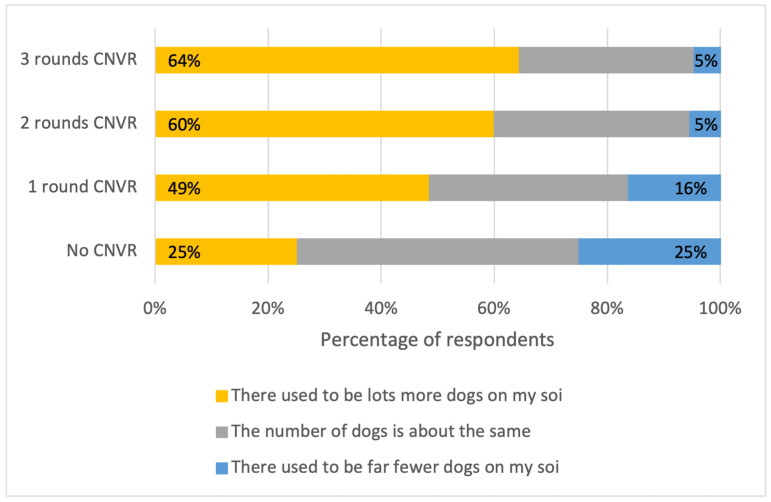
Percentages of respondents that selected each statement about changes in dog numbers on their soi from 9 years ago versus “today” (the day they were interviewed in 2023), split by the number of rounds of CNVR in their khet/subdistrict.

As with the perceived changes in street dog populations in a one-year timeframe, survey responses suggest a strong effect of the number of CNVR rounds. Respondents living in any subdistrict with CNVR had much higher odds of reporting a decrease in FRDs on their soi compared with nine years before. There was a stronger effect in areas with two or three rounds of CNVR, versus the control group, than in areas with one round (Table 3). Among respondents living in an area with three rounds of CNVR, 64% reported that there were more dogs on their soi nine years prior; only 5% reported the opposite. In the control areas without CNVR, 25% of respondents reported that there were more dogs nine years prior. An additional 25% reported fewer dogs in this timeframe (Figure 2).

Respondents were also asked how troubled they were by street dogs in 2023 compared with nine years prior. Of the 2998 responses, 31% (919) shared that they were less troubled currently, and 23% (691) reported equal levels of concern. Only 7% (195) indicated being more troubled by dogs now. The remaining 40% could not answer, as they either did not know or moved to the area recently.

**Figure 3:**
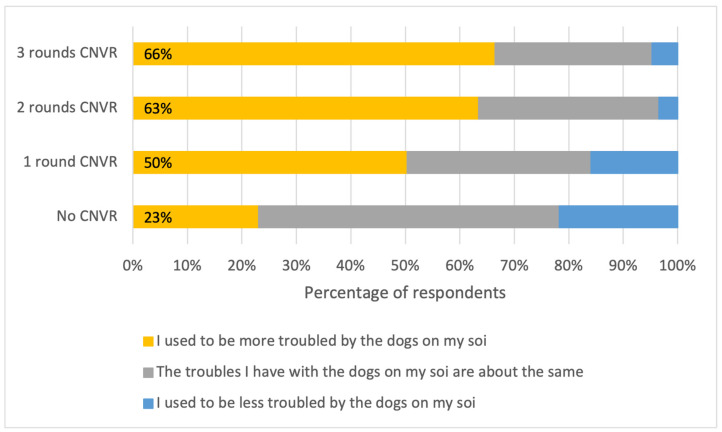
Percentages of respondents that selected each statement about trouble with FRDs on their soi 9 years ago compared with “today” (the day they were interviewed in 2023), split by the number of rounds of CNVR in their khet/subdistrict.

Respondents in a khet/subdistrict that had experienced any number of CNVR rounds had higher odds of reporting being more troubled by FRDs on their soi nine years prior (versus being equally or less troubled in the past) compared with those in the control areas (Table 4). Among respondents who lived in a subdistrict with any number of CNVR rounds, at least 50% felt less troubled now, and this rose to over 60% among those in areas with three CNVR rounds, compared to only 23% in control areas (Figure 3).

Dog owners had slightly (23%) lower odds than non-owners of reporting a decrease in the FRD numbers on their soi in the past year (Table 2). Ownership was not predictive of whether the respondents had perceived a change in FRD numbers, or experienced changes in how troubled they were by street dogs, compared with nine years ago (Table 3 and Table 4).

### 3.3. Puppies Born on Sois

Among 2974 responses, 6.7% (199 people) reported knowing of a litter of puppies born on their soi in the last 12 months. The majority, 93.4% (2775), did not know of puppies who were born. Respondents in khets/subdistricts that experienced any number of CNVR rounds had lower odds of reporting a litter than respondents that lived in control areas, and these odds were lower still in areas with two and three rounds of CNVR compared with controls (Table 5). Dog owners had 50% higher odds of reporting a litter on their soi than non-owners (Table 5).

Two hundred respondents shared the fate of locally born puppies. Most often they reported that puppies had been kept by the family (23%, 55 puppies), given away (22%, 53 puppies), or abandoned on the street (21%, 50 puppies). Respondents reported that 9% of puppies (21) died and 3% (6) were left at a location known for dumping dogs. The outcomes were unknown for 15% (35). No puppies were reported to have been sold, given to a shelter, or killed. Eight percent of respondents reported an “other” outcome but were not asked to elucidate.

### 3.4. Resident Attitudes Towards Street Dogs at Present

A series of questions gauged respondents’ opinions about the current number of street dogs on their soi, and the level of annoyance or concern associated with them.

When asked how many dogs were roaming on their soi at the time of the survey, 3077 persons answered. They reported a median of two dogs (IQR: 1–4; range: 0–34). The dimensions of their soi were not measured, so this number cannot be used to estimate a population size, but asking for it focused the respondent’s attention on their local population as they answered the next question. When asked how they viewed the number they had just described, 58% (1784) had a neutral response. An additional 35% (1088) had positive associations, reporting either that they “accept” or were “happy with” the number of dogs. The remaining 7% (205) had negative associations, reporting either that they “do not accept” or were “unhappy” with the number of dogs on their soi.

In khets/subdistricts with one round of CNVR, 39% of respondents reported being happy or accepting, and in those with two rounds, 45% of respondents reported being happy or accepting. Respondents that lived in khets/subdistricts with one or two rounds of CNVR had odds of being happy or accepting that were approximately twice those of respondents that lived in control areas. There was not a significant difference in reported happiness or acceptance between respondents that lived in areas with three rounds of CNVR and those that lived in control areas.

**Figure 4:**
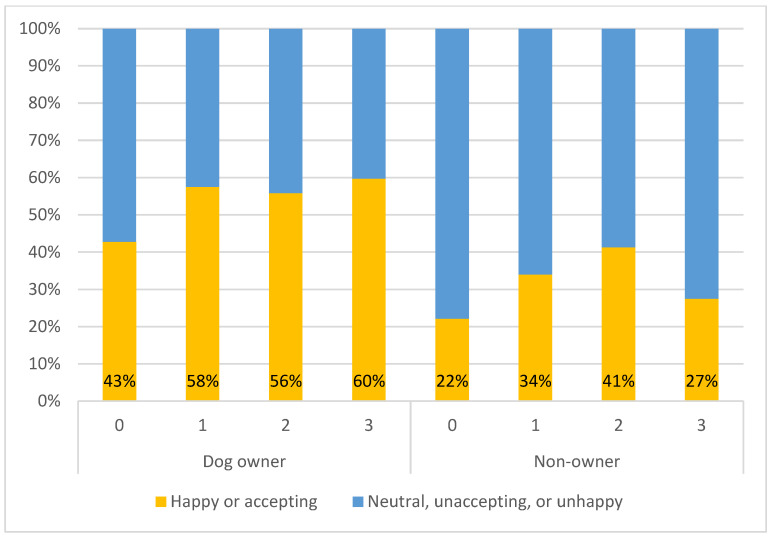
Percentages of respondents who reported being happy with or accepting of the number of FRDs on their soi compared with the combination of the other 3 categories (neutral, unaccepting, or unhappy), split by dog ownership and the number of CNVR rounds in their khet/subdistrict.

Dog ownership was correlated with attitudes towards FRDs. Dog owners had odds that were more than twice as high as non-owners of reporting that they were happy with or accepting of the number of FRDs on their soi (Table 6). At every level of CNVR, dog owners reported being happier with or accepting of these dogs (Figure 4).

Respondents were asked a related question of whether they had been annoyed or troubled by any dogs on their soi in the past month. Of the 3163 answers, 13.5% (426) were affirmative. The three most common reasons for being annoyed or troubled were noise (55%, 233), urine and faecal contamination (51%, 216), and dogs making a mess of garbage (46%, 196). In total, 16 percent (69) of respondents reported being chased by a dog, and 6% (24) reported that their pet had been attacked. Fewer than 5% of respondents cited destruction of property, dog bites, attacks on livestock, dog fights, damage to crops, concern for puppies, or sexual behaviour as causes for feeling annoyed or troubled, and no one selected a rabid, injured, or sick dog.

**Figure 5:**
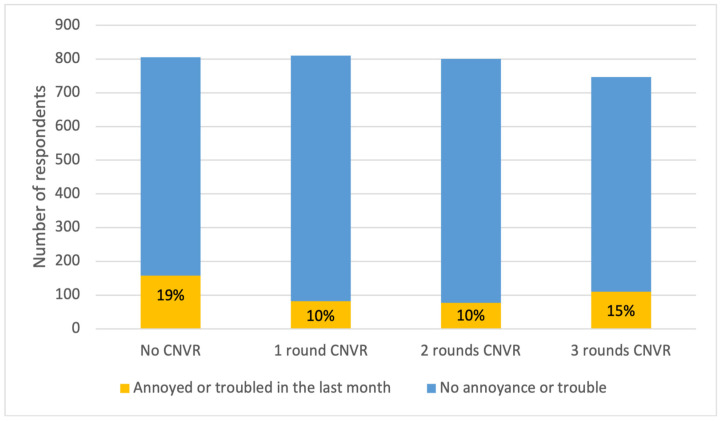
Numbers of respondents (data labels within bars show percentages) reporting that they were annoyed or concerned about FRDs within the previous month, split by the number of rounds of CNVR in their khet/subdistrict.

The number of CNVR rounds within a khet/subdistrict influenced the probability of being annoyed or troubled by a street dog (Figure 5). Respondents living in subdistricts with one or two rounds of CNVR had significantly lower odds of reporting annoyance or concern compared with those in control areas (Table 7). There was no significant difference in the odds of annoyance or trouble between respondents living in areas with three rounds of CNVR and those living in control areas. Dog ownership status was also not predictive (Table 7).

### 3.5. Care Offered to Street Dogs

When asked about providing care to street dogs in the past week, 14.3% (448) of the 3136 respondents had done so. Among this subset, the leading forms of care provided were food (95%, 443 people) and water (71%, 328). Fewer people reported offering shelter (12%, 54) or affection (7%, 31). Totals of 1% and 2% of respondents, respectively, reported taking a street dog to be vaccinated or sterilised in the past week.

Persons that lived in khets/subdistricts with two or three rounds of CNVR had lower odds of reporting that they had cared for a street dog in the last week versus those in control areas, with a highly significant difference between khets/subdistricts with two rounds of CNVR and control areas. There was no difference between khets/subdistricts with one round of CNVR and control areas. Dog owners had odds of caring for street dogs that were approximately 50% higher than the odds of non-owners (Table 8).

### 3.6. Dog Population Management Preferences

When asked what should be done to manage street dog numbers, 55% (1726) of 3133 respondents preferred sterilisation, and 20% (642) advocated for sheltering. Much smaller percentages advised rehoming or leaving the dogs alone (11% and 10%, respectively), and 4% of respondents did not know. Although 1 in 5 respondents recommended sheltering as the population management strategy, only 1.3% of respondents (38) reported that they had visited a Thai shelter.

Dog ownership, the number of rounds of CNVR in the khet/subdistrict, and prior experience of visiting a shelter were tested for their ability to predict whether a respondent preferred sterilisation (versus alternative approaches) or sheltering (versus alternative approaches) to address FRD populations. Respondents that lived in a khet/subdistrict with two CNVR rounds had over six times higher odds of selecting sterilisation, and 50% lower odds of selecting sheltering, as their preferred population management approach compared with respondents in control areas. Respondents in areas with three rounds of CNVR had odds of preferring sheltering that were nearly twice those of respondents in control areas. There was not a significant difference in their odds of selecting sterilisation. There was no difference in the respondents’ dog population management opinions in areas with one round of CNVR versus control areas (Table 9 and Table 10).

Dog ownership was a strong predictor of preference for population management strategy. Dog owners had over twice the odds of choosing sterilisation as their preferred management approach for FRDs, and significantly lower odds of choosing sheltering, compared with non-owners. A prior visit to a shelter in Thailand did not appear to affect respondents’ preferred population management approach (Table 9 and Table 10).

### 3.7. Public Health and Dog Bite Data

Of the 3164 persons who responded to a question about dog bites, only 1.5% (48) reported a dog bite in their household in the previous year. These 48 households reported 51 bites. Most bite victims were adults (86%) and male (59%). Approximately half of biting dogs were owned (51%) by either the victim’s (14%) or another (37%) household.

There were no significant differences in the odds of being bitten in a khet/subdistrict that had undergone one or two rounds of CNVR versus one that had not experienced any CNVR. However, the odds of reporting a bite in CNVR areas with three rounds were far lower than in the control areas (OR 0.27, 95% CI 0.07–1.06), although this was a statistical trend at *p* = 0.06. Dog ownership did not affect the odds of being bitten within the previous year.

## 4. Discussion

This study surveyed over 3200 residents of Greater Bangkok about their experiences, perceptions, and practices regarding FRDs on their soi and owned dogs in their household. This survey was one method used to evaluate an extensive initiative to sterilise and rabies-vaccinate FRDs in Greater Bangkok. It looked at the public’s attitudes and reported behaviours towards FRDs in relation to the number of CNVR rounds (ranging from 0 to 3) in their khet/subdistrict, as well as their dog ownership status. (Additional survey findings relating to owned dogs were reported by Hiby et al., 2025 [32]).

Survey responses suggest that CNVR significantly impacted residents’ observations of how FRD numbers have changed in their communities. Residents living in khets or subdistricts that had received CNVR attention were more likely than those in control areas (which did not receive CNVR) to report a decrease in the number of free-roaming dogs on their soi over the previous year compared with a landmarked point in time nine years prior, before the CNVR initiative began. Not only did residents of khets/subdistricts that experienced CNVR observe short- and long-term decreases in FRD numbers, but the results also show a consistent dose–response relationship: the more rounds of CNVR, the higher the proportion of people who perceived a reduction. This relationship was also evident for puppies born in the past year. As the number of CNVR rounds increased, the odds of a respondent reporting the birth of a litter on their soi decreased, a likely outcome of the higher sterilisation rates in the local area.

A dose–response relationship also existed with how “troubled” people were by dogs on their soi now versus nine years ago. As the number of CNVR rounds increased, so did the proportion of respondents who reported that they were more troubled by dogs in the past than at present. However, the relationship between rounds of CNVR and the experience of being “annoyed by” or “concerned about” FRDs in the preceding month was less consistent. Whereas residents of areas with one or two CNVR rounds had lower odds of reporting being annoyed with or concerned about FRDs in the previous month compared with the control areas, there was no difference in the odds between three rounds of CNVR and controls. Consistent with this finding, more residents of the areas that experienced one or two rounds of CNVR reported being “happy” with or “accepting” of the number of FRDs on their soi than residents in control areas. However, three rounds of CNVR did not further augment positive attitudes.

Across all CNVR levels, 14% of respondents reported offering care, predominantly food and water, to street dogs in the past week. The relationship between rounds of CNVR and care provisioning was unclear, but there appeared to be lower odds of reporting care provisioning to street dogs where CNVR had been delivered over at least two rounds, potentially because this was associated with a decline in the number of street dogs that required care.

The associations between CNVR and preferred population management approach were also notable. Survey respondents generally advocated for some intervention (versus taking no action), but which one varied with the number of rounds of CNVR that had occurred locally. Persons living in khets/subdistricts with two rounds of CNVR had over six times higher odds of preferring sterilisation, and 50% lower odds of preferring sheltering, compared with controls. This suggests a positive experience with CNVR. However, persons living in khets/subdistricts with three rounds of CNVR had odds nearly twice those of persons living in control areas of preferring sheltering, and no significant difference in odds of preferring sterilisation. The areas that had received three rounds of CNVR were likely to have experienced the largest declines in FRD populations [20]. This might have led residents to consider sheltering the remaining few street dogs as a feasible option, whilst residents living with larger local populations of street dogs would understand that sheltering them all would be unviable.

A key driver for CNVR is to reduce dog bites and rabies risk in communities with large numbers of FRDs. In this survey, only 1.5% of households reported experiencing a dog bite within the previous year, and about half of the bite victims reported that the offending dog was owned. The other half reported that the dog was (perceived to be) unowned. Although one and two rounds of CNVR did not have a statistically significant impact on bite incidence compared with the control areas, three rounds of CNVR did show a statistical trend towards a lower reporting of bites, with a relatively large effect size; only 0.5% of respondents reported bites from areas with three rounds of CNVR. It is curious that nearly 90% of reported bite victims were adults, and more than half were male. These findings do not align with other Thai studies, which found that most victims are children, and most biting dogs are “stray” or street dogs [39,40]. This disparity could potentially be explained by who receives medical care for dog bites in Thailand, with children more likely to be taken for medical attention than adults.

Dog ownership must be part of discussions about FRDs and CNVR. Among the Greater Bangkok residents who took part in this survey, 13.2% owned one or more dogs [32]. Dog owners were more aware of puppies born on their soi than were non-owners, and owning a dog appeared to positively influence a person’s attitudes towards FRDs. At every CNVR level (one, two, or three rounds), dog owners had higher odds of reporting being happy with or accepting of FRDs on their soi compared with non-owners. They had higher odds of caring for FRDs, a trend also identified elsewhere in Thailand [41]. Dog owners also had over twice the odds as non-owners of preferring sterilisation for managing FRD populations, and significantly lower odds of choosing sheltering. This latter option is impractical where there are large numbers of FRDs and minimal adoption from shelters (less than 1% of owned dogs in Bangkok were reported to have been adopted from a shelter [32]). Under such circumstances, shelters are associated with poor health and welfare for dogs, especially in cases of overcrowding or long duration of stay [42,43,44]. All these measures suggest that dog owners had a higher level of tolerance and care for FRDs. Furthermore, this tolerance has the potential to evolve into dogs being adopted into households; nearly half of owned dogs in Bangkok were reported to have been acquired via this route [32].

As reported in our parallel publication focusing on owned dogs [32], the keeping and care of the dogs belonging to these owners also appeared to be influenced by CNVR in meaningful ways. Owners living in areas with CNVR were less likely to allow their dogs to roam and more likely to have sterilised dogs. There was a dose–response relationship: as the number of CNVR rounds rose, so did the odds of sterilising and confining one’s dog. Higher sterilisation levels make sense given that CNVR mobile clinics treated owned dogs. Rabies vaccination levels were high for owned dogs overall, and there was no significant increase in the areas that experienced CNVR programming. This is presumably because the local government and private veterinarians offer vaccination, and so owners were not relying on the CNVR programme.

There is strong evidence that roaming owned dogs and abandoned owned dogs constitute a large proportion of FRDs in Greater Bangkok [20]. This is seen in other areas of Thailand, and in other countries [4,15,17,31]. It is therefore particularly important that CNVR efforts be coupled with interventions that focus on owned dogs. Depending on local attitudes, resources, and dog ownership practices, examples might include a microchip and registration system, controls on dog breeding or acquisition, promotion of responsible ownership, and readily accessible and affordable sterilisation services to avoid unwanted litters and breeding behaviours [7,9,32].

There are considerations and limitations to this study. Residents’ perceptions and behaviours were not tracked longitudinally and in response to the CNVR intervention, but retrospectively from a single point in time. The survey also worked with a distribution of CNVR effort across Greater Bangkok that was strategic with regard to operational roll out but not balanced with regard to potential unknown pre-existing human perceptions and behaviours. Dose-dependent effects of increasing CNVR rounds on several outcomes of interest indicate that CNVR was a driving factor. However, for some outcome measures, the khets/subdistricts with three rounds of CNVR were *less* successful than those with one or two rounds: their residents were less accepting of or happy with street dogs and preferred sheltering over sterilisation to manage FRD populations. There are confounding variables that could influence survey responses, including religion, ethnicity, socioeconomic status, degree of urbanisation, and housing type [45,46]. Even factors like ambient noise level could be relevant, especially given that noise was the most-reported reason for annoyance or trouble with FRDs. These variables all relate to where people live.

Without extending the questionnaire to include many demographic questions or adding pre- and post-intervention measures within the same household or khet/subdistrict, it is not possible to dismiss the possible impact of confounding variables on survey responses. That said, measures were taken to control for them. Respondents were recruited from across Greater Bangkok, and khet/subdistrict was included as a random effect in the statistical models. Including the khet/subdistrict improved the fit of the models, whereas including the province did not, indicating that if there are confounding variables that influence responses, they are not well captured simply by the province in which people reside.

An additional limitation was the consideration of the CNVR timing. The analysis accounted for the number of CNVR rounds in a khet/subdistrict, but not their timing. The most recent round of CNVR in a given community ranged from about one week to one year prior to conducting the survey. This study could not tease out whether or how the timing with which CNVR was conducted influenced peoples’ attitudes and behaviours towards street dogs, or their opinions of CNVR versus other methods of population control—for example, a recent CNVR intervention might make residents feel less troubled by FRDs because they felt that action was being taken to help. This is inevitable in a real-world study.

There was potential for both interviewer bias and social desirability bias in this survey. Regarding the former, interviewers knew how many rounds of CNVR had been conducted in each khet/subdistrict, and this could potentially create bias. However, only a marginal effect is likely from interviewer bias, and the effect sizes of CNVR are large for most outcomes of interest, suggesting that any impact would be inconsequential to the results. It is also possible that people gave responses that they thought the interviewer wanted to hear. This study’s design sought to reduce social desirability bias by not including Soi Dog Foundation in the consent statement, but in less than 10% of surveys, the respondent asked for and was told the interviewer’s affiliation. In those cases, it is possible that their responses were influenced by their perception of the Soi Dog Foundation and its mission. However, again, the impacts of CNVR on the outcomes of interest are mostly large effect sizes that are unlikely to have been produced by such biases.

## 5. Conclusions

The results of this survey indicate that CNVR is reducing FRD populations in Greater Bangkok at a level that is meaningful and noticed by residents, and is associated with improvements in residents’ experiences with, and tolerance of, FRDs. Most outcomes showed increasing benefits with increasing numbers of CNVR rounds, which is evidence of a dose-dependent effect of CNVR and suggests that CNVR, rather than a different underlying factor, is generating these changes. The benefits of CNVR reported by citizens should be compelling to policymakers considering investment in humane dog population management. Given the contribution of the owned dog population to FRDs, as evidenced in this study and others, effective humane dog population management strategies also require giving attention to dog owner practices and owned dog populations, including addressing causes of abandonment.

## Figures and Tables

**Table 1:** Total number of households (grey rows) and number of households in sample (white rows) split by province and by number of CNVR rounds.

		Province						
Number of CNVR Rounds	Households	Bangkok	Nakhon Pathom	Nonthaburi	Pathum Thani	Samut Prakan	Samut Sakhon	Total
0	Sample	0	0	129	684	0	0	813
	Total	0	0	36,674	235,736	0	0	272,410
1	Sample	0	194	230	172	226	0	823
	Total	0	137,501	703,175	441,310	587,873	0	1,869,860
2	Sample	31	551	0	0	32	191	807
	Total	449,760	288,704	0	0	45,644	146,057	930,167
3	Sample	419	0	0	0	89	255	766
	Total	2,748,105	0	0	0	116,905	163,766	3,028,779
Total	Sample	450	745	359	856	347	446	3203
	Total	3,197,865	426,205	739,849	677,046	750,422	309,823	6,101,210

**Table 2:** Predictors for the odds of a respondent reporting a decrease in the number of FRDs on their soi in the previous year versus perceiving that the number stayed the same or increased (binomial GLMM).

Respondent Reported a Decrease in the Number of FRDs on Their Soi	Coefficient	Odds Ratio	95% Confidence Interval	*p*-Value
1 round of CNVR vs. control (0 CNVR)	2.032	7.63	2.39–24.36	<0.001 ***
2 rounds of CNVR vs. control (0 CNVR)	3.155	23.46	7.30–75.34	<0.001 ***
3 rounds of CNVR vs. control (0 CNVR)	2.804	16.50	5.15–52.90	<0.001 ***
Dog-owning household vs. non-owner	−0.263	0.77	0.59–1.00	0.05 *

Significance: “***” < 0.001, “*” < 0.05.

**Table 3:** Predictors for the odds of a respondent reporting more FRDs on their soi 9 years ago versus the present, compared with perceiving the number of dogs staying the same or there being fewer in the past (binomial GLMM).

Respondent Reported That There Used to Be More FRDs on Their Soi 9 Years Ago	Coefficient	Odds Ratio	95% Confidence Interval	*p*-Value
1 round of CNVR vs. control (0 CNVR)	1.238	3.45	1.31–9.06	0.02 *
2 rounds of CNVR vs. control (0 CNVR)	1.991	7.32	2.92–18.32	<0.001 ***
3 rounds of CNVR vs. control (0 CNVR)	2.074	7.96	3.13–20.22	<0.001 ***
Dog-owning household vs. non-owner	−0.087			0.51

Significance: “***” < 0.001, “*” < 0.05.

**Table 4:** Predictors for the odds of a respondent reporting that they used to be more troubled by FRDs on their soi 9 years ago versus being equally or less troubled in the past (binomial GLMM).

Respondent Reported That They Used to Be More Troubled by FRDs on Their Soi 9 Years Ago	Coefficient	Odds Ratio	95% Confidence Interval	*p*-Value
1 round of CNVR vs. control (0 CNVR)	1.287	3.62	1.31–10.03	0.01 *
2 rounds of CNVR vs. control (0 CNVR)	2.119	8.32	3.21–21.60	<0.001 ***
3 rounds of CNVR vs. control (0 CNVR)	2.303	10.00	3.79–26.41	<0.001 ***
Dog-owning household vs. non-owner	−0.090			0.53

Significance: “***” < 0.001, “*” < 0.05.

**Table 5:** Predictors for the odds that a respondent knew of a litter of puppies born in a household on their soi within the last year (binomial GLMM).

Respondent Knew of a Litter of Puppies Born in a Household on Their Soi	Coefficient	Odds Ratio	95% Confidence Interval	*p*-Value
1 round of CNVR vs. control (0 CNVR)	−0.740	0.48	0.22–1.04	0.06 .
2 rounds of CNVR vs. control (0 CNVR)	−1.192	0.30	0.13–0.69	0.004 **
3 rounds of CNVR vs. control (0 CNVR)	−1.316	0.27	0.11–0.63	0.002 **
Dog-owning household vs. non-owner	0.417	1.52	1.07–2.15	0.02 *

Significance: “**” < 0.01, “*” < 0.05, “.” < 0.1.

**Table 6:** Predictors for the odds of a respondent reporting being happy with or accepting of the number of FRDs on their soi compared with the combination of the other 3 categories of neutral, unaccepting, or unhappy (binomial GLMM).

Respondent Was Happy with or Accepting of the Number of FRDs on Their Soi	Coefficient	Odds Ratio	95% Confidence Interval	*p*-Value
1 round of CNVR vs. control (0 CNVR)	0.541	1.72	0.95–3.10	0.07 .
2 rounds of CNVR vs. control (0 CNVR)	0.832	2.30	1.28–4.13	<0.01 **
3 rounds of CNVR vs. control (0 CNVR)	0.191			0.53
Dog-owning household vs. non-owner	0.837	2.31	1.86–2.87	<0.001 ***

Significance: “***” < 0.001, “**” < 0.01, “.” < 0.1.

**Table 7:** Predictors for the odds of a respondent reporting being annoyed or concerned about FRDs on their soi in the previous month (binomial GLMM).

Respondent Was Annoyed or Concerned About FRDs in the Previous Month	Coefficient	Odds Ratio	95% Confidence Interval	*p*-Value
1 round of CNVR vs. control (0 CNVR)	−0.694	0.50	0.29–0.87	0.01 *
2 rounds of CNVR vs. control (0 CNVR)	−1.037	0.35	0.20–0.63	<0.001 ***
3 rounds of CNVR vs. control (0 CNVR)	−0.296			0.28
Dog-owning household vs. non-owner	−0.151			0.29

Significance: “***” < 0.001, “*” < 0.05.

**Table 8:** Predictors for the odds of a household providing care to a street dog(s) within the previous week (binomial GLMM).

Household Provided Care to a Street Dog(s) Within the Previous Week	Coefficient	Odds Ratio	95% Confidence Interval	*p*-Value
1 round of CNVR vs. control (0 CNVR)	0.036			0.93
2 rounds of CNVR vs. control (0 CNVR)	−1.222	0.29	0.12–0.69	0.005 **
3 rounds of CNVR vs. control (0 CNVR)	−0.823	0.44	0.19–1.03	0.06 .
Dog-owning household vs. non-owner	0.382	1.47	1.12–1.91	0.005 **

Significance: “**” < 0.01, “.” < 0.1.

**Table 9:** Predictors for the odds of a respondent selecting sterilisation as their preferred approach to dog population management compared with the combination of shelter, rehoming, or leaving dogs alone (binomial GLMM).

Respondent Selected *Sterilisation* as Their Preferred Dog Population Management Approach	Coefficient	Odds Ratio	95% Confidence Interval	*p*-Value
1 round of CNVR vs. control (0 CNVR)	0.276			0.39
2 rounds of CNVR vs. control (0 CNVR)	1.895	6.65	3.48–12.70	<0.001 ***
3 rounds of CNVR vs. control (0 CNVR)	0.423			0.19
Dog-owning household vs. non-owner	0.867	2.38	1.88–3.01	<0.001 ***
Visited a shelter in Thailand vs. not visited	0.224			0.57

Significance: “***” < 0.001.

**Table 10:** Predictors for the odds of a respondent selecting sheltering as their preferred approach to dog population management compared with the combination of sterilisation, rehoming, or leaving dogs alone (binomial GLMM).

Respondent Selected *Shelter* as Their Preferred Dog Population Management Approach	Coefficient	Odds Ratio	95% Confidence Interval	*p*-Value
1 round of CNVR vs. control (0 CNVR)	0.004			0.99
2 rounds of CNVR vs. control (0 CNVR)	−0.0659	0.52	0.28–0.95	0.03 *
3 rounds of CNVR vs. control (0 CNVR)	0.589	1.80	1.01–3.22	0.05 .
Dog-owning household vs. non-owner	−0.864	0.42	0.31–0.57	<0.001 ***
Visited a shelter in Thailand vs. not visited	−0.497			0.34

Significance: “***” < 0.001, “*” < 0.05, “.” < 0.1.

## Data Availability

The data presented in this study are available upon request from the corresponding author. The data are not publicly available due to the consent agreement with the participants.

## Supplementary Materials

The following supporting information can be downloaded from https://www.mdpi.com/article/10.3390/ani15091274/s1: The complete survey questionnaire is available in Supplementary File S1. Some questions were answered as “yes” or “no” to expose all subsequent follow-up questions that are dependent on these answers.
